# Behavioral changes and hygiene practices of older adults in Japan during the first wave of COVID-19 emergency

**DOI:** 10.1186/s12877-021-02085-1

**Published:** 2021-02-24

**Authors:** Yasumichi Arai, Yuko Oguma, Yukiko Abe, Midori Takayama, Azusa Hara, Hisashi Urushihara, Toru Takebayashi

**Affiliations:** 1 Centre for Supercentenarian Medical Research, Keio University School of Medicine, 35 Shinanomachi, Shinjuku-ku, Tokyo, 160-8582 Japan; 2 Sports Medicine Research Center, Keio University, 4-1-1 Hiyoshi, Kohoku-ku, Yokohama-shi, Kanagawa 223-8521 Japan; 3 Department of Foreign Languages and Liberal Arts, Faculty of Science and Technology, Keio University, 4-1-1 Hiyoshi, Kohoku-ku, Yokohama-shi, Kanagawa 223-8521 Japan; 4 Division of Drug Development and Regulatory Science, Faculty of Pharmacy, Keio University, 1-5-30 Shibakoen, Minato-ku, Tokyo, 105-8512 Japan; 5 Department of Preventive Medicine and Public Health, Keio University School of Medicine, 35 Shinanomachi, Shinjuku-ku, Tokyo, 160-85820 Japan

**Keywords:** Older adults, Behavior, Hygiene practice, COVID-19

## Abstract

**Background:**

On April 7, 2020, Japan declared a state of emergency due to the first wave of coronavirus disease 2019 (COVID-19) with the associated social distancing likely to have had a great impact on older adults’ lifestyle and health. This study aimed to explore the behavioral changes and personal hygiene practices in relation with background psychosocial and health characteristics of older adults during the COVID-19 emergency.

**Methods:**

A cross-sectional telephonic survey was conducted with the participants of the Kawasaki Aging and Wellbeing Project (KAWP), an on-going longitudinal cohort study of older adults aged 85 or older. The interviews were conducted using a structured questionnaire consisting of 11 closed questions regarding behavioral changes and personal hygiene practices during the state of emergency. Sociodemographic and health data were obtained from the KAWP baseline survey conducted 2.2 years before the telephonic survey.

**Results:**

Overall, 487 participants from the KAWP responded to the telephonic survey (response rate: 89.2%). 94.5% of the respondents reported no changes in basic lifestyle habits, such as eating, sleeping, smoking, and drinking, whereas 28.1% reported a decrease in physical activity, and 54.6% reported going out less frequently. One-third of the respondents reported a decrease in the number of people to converse with, as well as the amount of time to converse. For personal hygiene practices, 93.8% reported wearing a mask when they went out, and 50.3% reported an increased frequency of handwashing. Multiple logistic regression analysis revealed that engagement in physical activity at baseline (odds ratio [OR] = 1.95, 95% confidence interval [CI] = 1.23–3.08), smartphone ownership (OR = 2.15, 95% CI = 1.33–3.47), and visual impairment (OR = 1.79; 95% CI = 1.10–2.91) were independently associated with decreased physical activity during the COVID-19 emergency. Female respondents and smartphone ownership were significantly associated with more frequent handwashing.

**Conclusions:**

The study revealed that older adults in an urban setting responded to the COVID-19 emergency with behavioral changes. The findings of this study have implications for the design of preventive strategies to maintain the health and wellbeing of at-risk older adults.

**Supplementary Information:**

The online version contains supplementary material available at 10.1186/s12877-021-02085-1.

## Background

Since the first case of the novel coronavirus disease 2019 (COVID-19) was reported in Wuhan, China, in December 2019, the outbreak of COVID-19 has emerged as a global health emergency [1]. Older adults, particularly those with preexisting comorbidities, are at greater risk for severe COVID-19 outcomes, including hospital admission and death than others. Given the high mortality rate among those aged 80 years or older [2] and lack of evidence-based treatment, social distancing is of prime importance for preventing the spread of the severe acute respiratory syndrome coronavirus 2 (SARS-CoV-2) among the older population. However, social distancing can lead to both physical inactivity and social isolation. These create concerns about the increasing risks of sarcopenia, depression, and other chronic diseases of aging [3].

High case fatality rate and uncertainties about the convergence of COVID-19 outbreaks necessitate an urgent need to establish a practical method to prevent SARS-CoV-2 transmission and maintain the health and wellbeing of the older adults. On April 7, 2020, Japan declared a state of emergency in seven prefectures, including the Greater Tokyo Area [4]. It aimed to reduce social contact among people by 80% and continued it until May 25, 2020. With some countries experiencing the third wave of COVID-19 cases starting from November 2020, Japan declared the second state of emergency for Tokyo and its neighboring prefectures on 7 January 2021 [5]. Social distancing and stay-at-home order for extended periods is a serious public health concern as it can increase the risk of adverse mental and sedentary health outcomes, particularly in a vulnerable population, such as very old people and those who are not technology-driven [6]. Therefore, the aims of the present study were 1) to examine self-reported impacts of the COVID-19 emergency on basic lifestyle, physical activity, and personal hygiene practices, and 2) to identify subgroups of older adults who might be more susceptible to the negative impact of COVID-19 emergency using an on-going longitudinal cohort of older adults independently living in an affected community.

## Methods

### Study population

The Kawasaki Aging and Wellbeing Project (KAWP) is a longitudinal cohort study of older adults aged between 85 and 89 with no physical disability at baseline. The prime aim of the KAWP is to explore trajectories of functional decline, frailty, and cognitive impairment, and to identify genetic, biological, behavioral, and socioenvironmental factors that delay or modify this deteriorating process at an advanced age. The inclusion criteria of KAWP are: 1) being a resident of Kawasaki city, a city with a population of 1.5 million, located in the Greater Tokyo Area and aged between 85 and 89; 2) having no limitations in the basic activities of daily living (ADL); and 3) being able to visit the study site, the Kawasaki Municipal Hospitals independently.

**Fig. 1:**
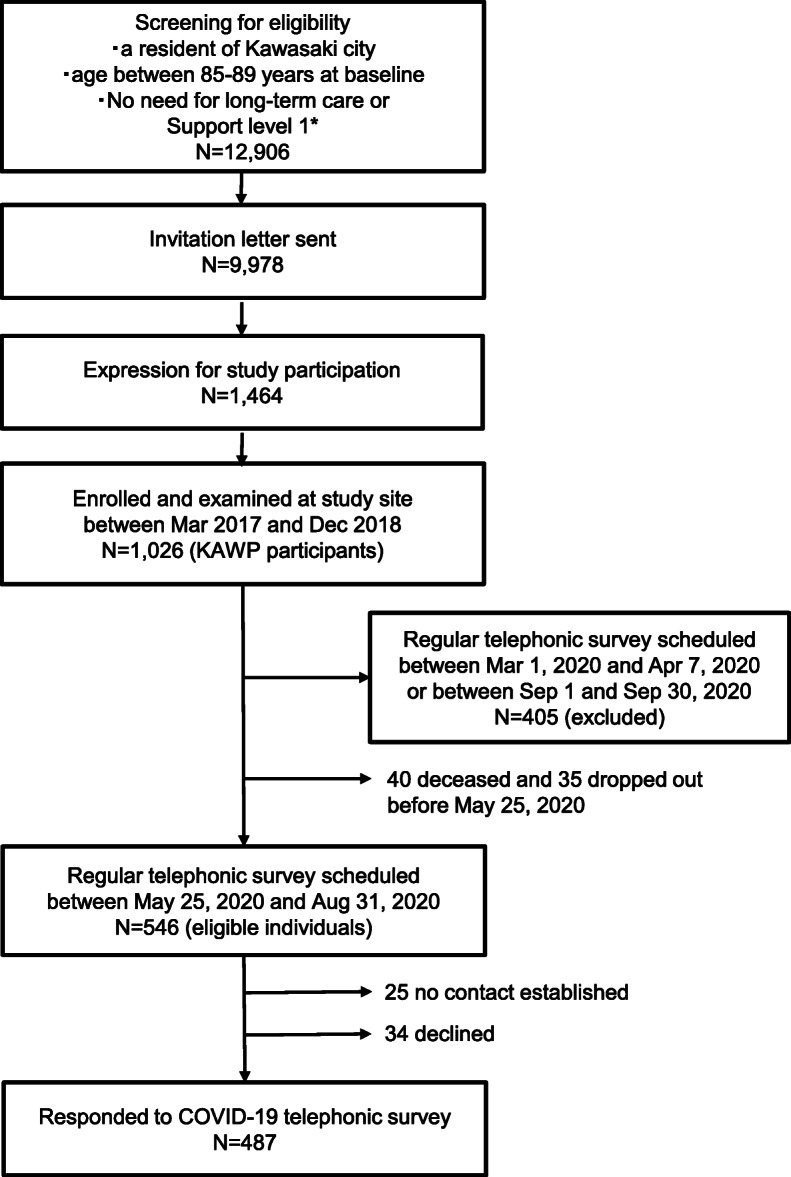
Flowchart of the study recruitment. * There are seven categories of the long-term care benefits and support in Japan: No certified (no need for long term care), support levels 1& 2 for preventive long-term care benefits, care levels 1 to 5 for long-term care benefits. The higher the level of care, the more advanced the functional decline

Using the basic registration of residents and the long-term care insurance database, a total of 12,906 were screened as potential participants. Among them, we mailed an invitation letter for this study to 9978 individuals, and 1464 eligible residents responded to express their willingness to participate in the study. Between March 2017 and December 2018, a total of 1026 independent seniors were enrolled in the KAWP and a comprehensive baseline assessment, including assessment of physical, mental, and cognitive function as well as social participation was conducted (Fig. 1). Thereafter, the participants were scheduled for telephonic surveys every 6 months to monitor their vital status, any incidental disabilities, falls and fractures, and hospitalizations until December 2024 or until they dropped out.

In this study, we selected 546 people who were scheduled for follow-up telephonic surveys between May 25, 2020, the day the state of emergency was lifted, and the end of August 2020. Written informed consent to participate in the KAWP was obtained from all participants. The KAWP was approved by the ethics committee of the Keio University School of Medicine (ID: 20160297) and was registered in the University Hospital Medical Information Network Clinical Trial Registry as an observational study (ID: UMIN000026053).

### Measurements

#### COVID-19 questionnaire

The COVID-19-related telephonic interviews were conducted by two trained interviewers between May 25 and August 31, 2020 using a structured questionnaire (Additional file 1) in addition to the usual 6-month follow-up telephonic survey. The questionnaire consisted of 11 closed-ended questions and one open-ended question regarding perceived changes in basic lifestyle, physical activity, conversation time, and precautious behaviors during the state of emergency. Each interview was completed in approximately 10–20 min.

### Baseline characteristics

Sociodemographic and health data were obtained from the KAWP baseline survey conducted between March 2017 and December 2018 (on average 2.2 years before the telephonic survey). All participants were invited to visit one of three Kawasaki Municipal Hospitals (Kawasaki, Ida, or Tama) and were interviewed and examined using a study protocol that was harmonized with the Tokyo Oldest Old Survey on Total Health and Japan Semi-supercentenarian Study, both of which are managed by the Center for Supercentenarian Medical Research, Keio University School of Medicine [7, 8]. Participants were asked to fill out a pre-mailed questionnaire including education (high school and higher education or not), living situation, marital status, current alcohol use, smoking status, and self-rated health. They were checked for consistency by interviewers at the time of the baseline survey.

The medical interview was conducted by trained physicians, and the number of chronic conditions was counted based on past and present medical history of 18 diseases: cerebrovascular disease, cardiac disease, hypertension, diabetes, dyslipidemia, respiratory disease, gastrointestinal disease, renal disease, prostate disease, thyroid disease, Parkinson’s disease, connective tissue disease, eye disease, osteoporosis, arthritis, hyperuricemia, malignancy, and dementia. Body weight and height were measured while wearing light clothes and standing upright. Instrumental activities of daily living (IADL) were assessed using the Lawton scale (0–5 points) [9], cognitive functions were evaluated according to the Mini-Mental State Examination (MMSE; 0–30 point) [10] and depression was assessed using the Geriatric Depression Scale (GDS-15) [11]. Self-rated health is scored by a Likert-type scale ranging from 1 (very poor) to 5 (very good). Physical activity in the past 1 year was assessed using the modified Zutphen Physical Activity Questionnaire [12]. The questionnaire determined the frequency and duration of walking, cycling, and other leisure-time physical activities. Physical Activity Index (PAI) was then calculated by multiplying the activity intensity (compendium-coded, metabolic equivalents, METs) with duration (hours) and frequency (times per week). The questionnaire was validated with a tri-axial accelerometer and physical functions in the very old adults [12]. Upper tertile of PAI was regarded as engagement in physical activity. Hearing and visual acuity were rated according to self-reported categories. Those who responded “need a loud voice or speak in his/her ears” or “cannot hear at all” were judged as having a hearing impairment. Those who rated “poor” and “very poor” for eyesight were regarded as visually impaired. Self-reported community interaction was evaluated according to the following question: “How often do you meet or talk to people in the community you associate with (including phone and email exchanges)?” Here, a community is defined as an area that is about a 10-min walk away.

### Statistical analysis

Baseline characteristics are expressed as medians and interquartile ranges (IQR); categorical variables are shown as numbers and proportions. Correlation between numerical variables assessed at baseline survey was calculated with Spearman’s correlation coefficients. Crude odds ratio (OR) and 95% confident interval (CI) were calculated for behavioral factors such as decreased physical activity and conversation time and washing hands more. Multivariable logistic regression analysis was performed to examine the independent association between behavioral factors and baseline characteristics, in which all variables were mutually adjusted in the models. All analyses were performed using SPSS Statistics ver. 24.0 software (Armonk, NY: IBM Corp.), and results were considered statistically significant at a *P* -value of < 0.05, and two-sided tests were applied.

## Results

Of the 546 participants eligible for the telephonic survey during the study period, 25 were not contactable, 34 declined (7 were institutionalized, 11 had hearing problems, 7 had poor health, 3 did not consent, 5 had family reasons, and 1 had unknown reasons); thus, 487 respondents completed the telephonic questionnaire (Fig. 1, response rate: 89.2%). The median age of the respondents was 89.3 (IQR: 88.3–90.6) years. More than half of the respondents were women (50.5%), and 45.0% of the respondents were widowed, and 47.4% had high school or higher education (Table 1). The median MMSE score was 26 (IQR: 24–28), and 15.8% had MMSE scores≤23. Only 12.7% of the respondents had a 1 ≥ IADL disability, whereas the majority were IADL independent. There were no significant differences in basic characteristics between participants and non-participants in the telephone survey (Supplemenaty Table 1).

**Table 1:** Characteristics of the respondents

Age at telephone survey, (IQR)	89.3	(88.3–90.6)
Time since basic survey, years. (IQR)	2.2	(1.7–3.0)
Female, n. (%)	246	(50.5)
Living alone^a^, n. (%)	130	(26.7)
Marital status^a^, n. (%)		
Married	246	(50.5)
Widowed	219	(45.0)
Divorce	11	(2.3)
Never married	8	(1.6)
Missing	3	(0.6)
High education^a^, n. (%)	231	(47.4)
Missing	2	(0.4)
Smoking status^a^, n (%)		
Current smoker	21	(4.3)
Former smoker	182	(37.4)
Never smoker	281	(57.7)
Missing	3	(0.6)
Current alcohol intake^a^, n (%)		
No	292	(60.0)
Yes	194	(39.8)
Missing	1	(0.2)
BMI category^a^, n (%)		
Underweight	28	(5.7)
Normal weight	331	(68.0)
Overweight	116	(23.8)
Obesity	12	(2.5)
Self-rated health^a^, n. (%)		
Very/good	202	(41.5)
Fair	216	(44.4)
Poor/very poor	66	(13.6)
Don’t know	3	(0.6)
MMSE^a,^ (IQR)	26	(24–28)
MMSE^a^ (≤23)	77	(15.8)
Missing	3	(0.6)
GDS^a^, (IQR)	3	(1–4)
GDS^a^ (≥5)	119	(24.4)
Missing	3	(0.6)
IADL^a^, (IQR)	5	(5)
IADL^a^ (≤4)	62	(12.7)
Hearing impairment^a^, n. (%)	36	(7.4)
Missing	5	(1.0)
Visual impairment^a^, n. (%)	119	(24.4)
Missing	6	(1.2)
Number of chronic conditions^a^, n (%)		
0–1	25	(5.2)
2–4	219	(45.0)
5≤	234	(48.0)
Missing	9	(1.8)
Physical Activity Index^a^ (IQR), METs h/w	16.3	(9.7–27.8)
Missing	1	(0.2)
Frequency of community interaction^a^, n. (%)		
None	82	(16.8)
Several times/year	21	(4.3)
Several times/month	106	(21.8)
Several times/week	190	(39.0)
Everyday	63	(12.9)
Not sure	22	(4.5)
Missing	3	(0.6)

*Abbreviations*: *IQR* Interquartile range, *MMSE* Mini-Mental State Examination, *GDS* Geriatric Depression Scale, *IADL* Instrumental activities of daily living, *METs h/w* Metabolic equivalents hour per week

^a^ assessed at baseline survey

At baseline, amount of physical activity was associated with self-rated health and IADL (Table 2, Spearman’s rho = 0.126, *P* < 0.001; rho = 0.176, *P* < 0.001, respectively), while negatively associated with GDS and number of chronic conditions (rho = − 0.216, *P* < 0.001; rho = − 0.106, *P* < 0.05, respectively). Self-rated health was negatively associated with GDS and the number of chronic conditions (rho = − 0.166, *P* < 0.001; rho = − 0.179, *P* < 0.05, respectively).

**Table 2:** Spearman's rho correlation coeficients  for numerical measures

	Physical Activity Index	Self-rated health	MMSE	GDS	IADL
Self-rated health	.126***				
MMSE	.013	.014			
GDS	−.216***	−.166***	−.194***		
IADL	.176***	.096*	.123**	−.186***	
Number of chronic conditions	−.106*	−.179***	.081	.121**	−.075

Abbreviations: *MMSE* Mini-Mental State Examination, *GDS* Geriatric Depression Scale, *IADL* Instrumental activities of daily living. * *P* < 0.05, ** *P* < 0.01, *** *P* < 0.001

**Fig. 2:**
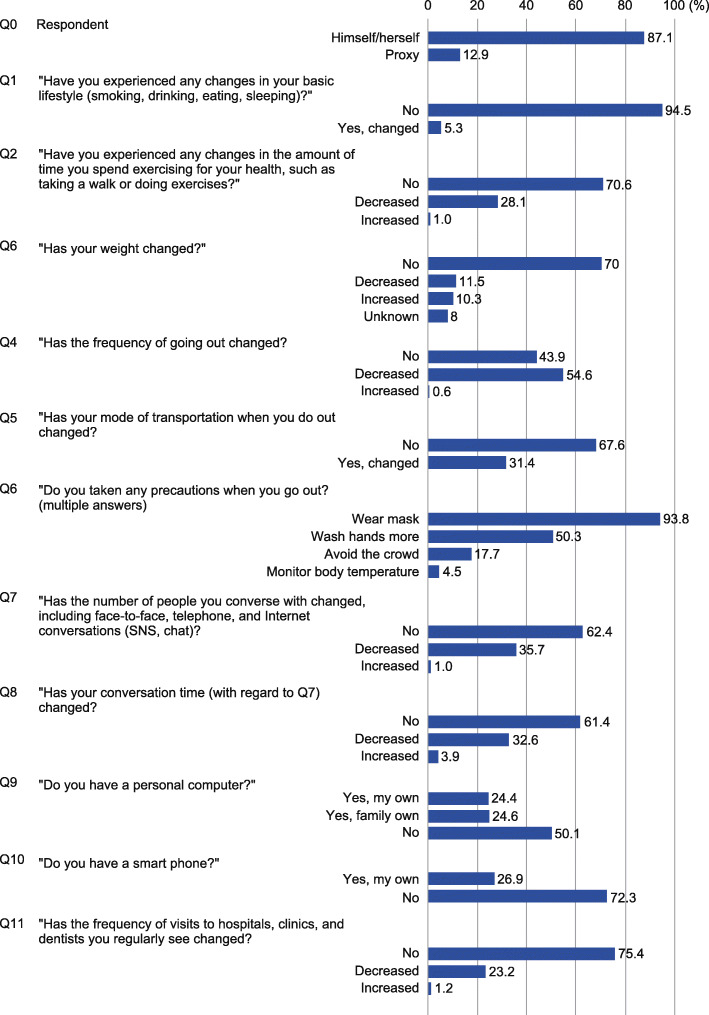
Behavioral changes and personal hygiene practice during the state of emergency due to COVID-19 outbreak. The number of missing values from Q1 to Q11 are 1 (0.2%), 1 (0.2%), 1 (0.2%), 4 (0.8%), 5 (1.0%), 4 (0.8%), 4 (0.8%), 10 (2.0%), 4 (0.8%), 4 (0.8%), 1 (0.2%), respectively

Figure 2 shows the responses to the survey questions regarding behavioral changes and personal hygiene practices during the COVID-19 emergency. Among the responders, 424 (87.1%) were interviewed directly, whereas 63 (12.9%) responded by proxy. Overall, most respondents (94.5%) reported no basic lifestyle changes, such as eating, sleeping, smoking, and drinking, whereas 28.1% reported a decrease in the amount of physical activity, and more than half (54.6%) reported reduced frequencies of going out. Similar percentages of respondents reported a decrease in the number of people to converse with and a decreased amount of time to converse (35.7 and 32.6%, respectively). For personal hygiene practice, 93.8% reported wearing a mask when they went out, whereas only 50.3% reported washing hands more often, and a few (4.5%) monitored their body temperature.

Multiple logistic regression analysis revealed that engagement in physical activity at baseline (OR = 1.95; 95% CI = 1.23–3.08), smartphone ownership (OR = 2.15; 95%CI = 1.33–3.47), and visual impairment (OR = 1.79; 95%CI = 1.10–2.91) were independently associated with a decreased amount of physical activity during COVID-19 emergency (Table 3). Engagement in physical activity at baseline was associated with decreased conversation time with neighbors (OR = 2.24; 95%CI = 1.43–3.50), while frequent community interaction was negatively associated with decreased conversation time (OR = 0.23; 95%CI = 0.10–0.55) as well as decreased number of conversation partners (Supplementary Table 2). Being women and smartphone ownership were significantly associated with more frequent hands-washing (Table 3). None of the baseline characteristics were independently associated with decreased clinic/hospital/dentists visit (Supplementary Table 2).

**Table 3:** Factors associated with decreased physical activity and conversation time, and enhanced handwashing

	Decreased physical activity				Decreased conversation time				Washing hands more			
	Crude		Adjusted^d^		Crude		Adjusted^d^		Crude		Adjusted^d^	
Characteristics	OR	95%CI	OR	95%CI	OR	95%CI	OR	95%CI	OR	95%CI	OR	95%CI
Age	0.97	(0.84–1.12)	0.95	(0.80–1.13)	0.86	(0.75–1.00) ^e^	0.85	(0.72–1.00)	0.99	(0.87–1.13)	1.02	(0.88–1.19)
Sex (female)	1.23	(0.83–1.83)	1.18	(0.69–2.02)	1.09	(0.74–1.59)	1.07	(0.64–1.80)	1.72	(1.20–2.47)^e^	1.90	(1.17–3.08)^e^
High education	0.77	(0.52–1.15)	0.69	(0.43–1.10)	1.01	(0.69–1.48)	0.97	(0.62–1.51)	1.26	(0.88–1.80)	1.44	(0.95–2.18)
Living alone	1.03	(0.66–1.61)	0.96	(0.56–1.65)	0.92	(0.60–1.42)	0.86	(0.51–1.44)	1.40	(0.93–2.10)	1.24	(0.76–2.03)
Widowed	1.15	(0.77–1.71)	1.09	(0.65–1.85)	1.10	(0.75–1.61)	1.25	(0.75–2.07)	1.30	(0.91–1.87)	1.08	(0.67–1.73)
Current alcohol use	1.15	(0.77–1.72)	1.35	(0.86–2.13)	0.95	(0.65–1.41)	1.02	(0.65–1.59)	0.93	(0.65–1.34)	0.97	(0.64–1.48)
Current smoker	0.59	(0.19–1.78)	0.67	(0.17–2.57)	0.78	(0.30–2.06)	0.69	(0.20–2.36)	0.46	(0.18–1.17)	0.76	(0.26–2.18)
Overweight	0.99	(0.63–1.55)	1.19	(0.73–1.94)	0.89	(0.58–1.37)	1.12	(0.70–1.82)	1.26	(0.84–1.89)	1.19	(0.76–1.86)
Self-rated health^a^	0.97	(0.65–1.45)	0.91	(0.58–1.44)	0.71	(0.48–1.05)	0.73	(0.47–1.13)	1.30	(0.90–1.87)	1.09	(0.73–1.64)
MMSE (≤23)	0.58	(0.31–1.05)	0.56	(0.29–1.08)	0.57	(0.32–1.01)	0.53	(0.28–1.01)	0.98	(0.60–1.61)	1.02	(0.59–1.77)
GDS (≥5)	0.78	(0.49–1.26)	0.79	(0.46–1.36)	1.16	(0.75–1.80)	1.12	(0.68–1.85)	0.71	(0.47–1.07)	0.93	(0.58–1.48)
IADL (≤4)	1.36	(0.77–2.39)	1.64	(0.85–3.19)	1.09	(0.61–1.92)	1.14	(0.59–2.23)	0.89	(0.52–1.52)	0.79	(0.43–1.46)
Chronic conditions ≥5	0.91	(0.61–1.35)	0.84	(0.54–1.30)	0.93	(0.63–1.37)	0.95	(0.62–1.45)	1.13	(0.79–1.62)	1.05	(0.71–1.57)
Hearing impairment	1.28	(0.62–2.63)	0.97	(0.42–2.26)	0.98	(0.48–2.01)	0.87	(0.38–1.98)	1.09	(0.55–2.16)	1.08	(0.50–2.30)
Visual impairment	1.74	(1.12–2.71)^e^	1.79	(1.10–2.91)^e^	1.28	(0.83–1.98)	1.18	(0.73–1.91)	1.05	(0.69–1.60)	1.28	(0.81–2.02)
Engagement in physical activity^b^	1.77	(1.17–2.67)^e^	1.95	(1.23–3.08)^e^	1.86	(1.25–2.76)^e^	2.24	(1.43–3.50)^e^	1.09	(0.75–1.60)	1.04	(0.68–1.58)
Frequent community interaction^c^	0.69	(0.37–1.29)	0.69	(0.34–1.40)	0.30	(0.15–0.63)^e^	0.23	(0.10–0.55)^e^	1.65	(0.96–2.84)	1.91	(1.03–3.55)^e^
Smartphone ownership	2.02	(1.32–3.10)^e^	2.15	(1.33–3.47)^e^	0.96	(0.62–1.48)	0.87	(0.54–1.41)	1.58	(1.05–2.37)^e^	1.81	(1.15–2.85)^e^

*Abbreviations*: *OR* Odds ratio, *CI* Confidence interval, *MMSE* Mini-Mental State Examination, *GDS* Geriatric Depression Scale, *IADL* Instrumental activities of daily living

^a^ Those who reported very good/good versus others (reference)

^b^ Those who reported upper tertile of physical Activity Index versus others (reference)

^c^ Those who reported every day versus others (reference)

^d^ Mutually adjusted for all variables in the model

^e^ *P* < 0.05

## Discussion

In this study of the very elderly people (aged around 90), we explored self-perceived behavioral changes and hygiene practices during the first wave of COVID-19 emergency in Japan. We examined the associations between these behavioral changes and demographic, psychosocial, and functional characteristics assessed before the COVID-19 outbreak. The majority of the older individuals went out less frequently during the declared state of emergency, likely because they adhered to the stay-at-home advice applicable to other age groups [4]. Previous studies reported a decline in physical activity during COVID-19 lockdown in various populations and countries. An online survey of 1491 Australian adults (mean age 50.5 ± 14.9 years) demonstrated that 48.9% of respondents report decreased physical activity associated with higher depression, anxiety, and stress symptoms [13]. In the Longitudinal Aging Study Amsterdam, which enrolled 1119 elderly (mean age 74 ± 7 years) to a self-administered questionnaire survey, Visser et al. demonstrated that about half of the respondents reported a decrease in physical activity. Older adults were less likely to report a negative impact on their physical activity or exercise levels than younger people [14]. They also reported that functional limitation was strongly associated with a negative impact on physical activity. The difference in the impact of COVID-19 outbreak on physical activity behavior might be partly accounted for by the characteristics of respondents (e.g., age, functional status). In this present study of the very old adults independently living in the community, 28.1% of respondents reported decreased physical activity, which is independent of limitation in IADL or depressive symptoms (high GDS score). In contrast, physical activity engagement at baseline and smartphone ownership were independently associated with reduced physical activity during the COVID-19 emergency. These two measures were also associated with more frequent hand washing, suggesting that reductions in physical activity in our older participants may be part of preventive behaviors to reduce social contact.

Individuals with health literacy but lost resources (e.g., day center, gym, group activities) can maintain their previous physical activity levels if alternative resources, such as smartphone apps and home-exercise programs, are provided. In this study, visual impairment is another factor associated with decreased physical activity during COVID-19 emergency, suggesting that visual impairment could be a barrier to (in)formal support and resources to exercise or maintain physical activity. Therefore, alternative tools should be considered with adaptations for people with visual impairment, who are more likely to be vulnerable to the negative impacts of CODID-19 emergency.

Our results did not show any association of IADL disability or cognitive impairment with decreased physical activity during the COVID-19 emergency. This might be partly because our cohort includes relatively small percentages of those with dementia (2.1%) and disability in IADL (12.7%) due to our inclusion criteria. Alternatively, since frail older adults may already have been physically inactive, this questionnaire may have minimal effect, and hence, long-term effects on physical and mental health should be cautiously monitored by future follow-ups.

To prevent the spread of COVID-19 infection, social distancing is the most visible public health measures, but its implications for mental health and wellbeing should be addressed in research, policy, and clinical approaches to the COVID-19 pandemic [15]. In this context, it is noteworthy that our results show that the elderly who had established active social relations in the community before COVID-19 and interacted with each other frequently are likely to maintain their social networks even during COVID-19 emergency. In recent years, a good social environment (e.g., social support, social networks, and social inclusion), as well as the physical environment, have been shown to affect the well-being and health of the elderly [16]. Our results suggest that having a good social environment in the community daily may prevent the decline and deterioration of the well-being and mental health of the elderly even in emergencies such as the COVID-19 pandemic. This notion will be addressed in future follow-up studies with a direct assessment of psychosocial health and wellbeing concerning the social network in our cohort.

Compared to the high compliance with wearing a mask while going out (93.8%), hand wash hygiene was relatively neglected (50.3%) by our older respondents. In an internet survey aimed at 3301 adults in the United States of America aged ≥18 in the 2015–2016 influenza season, washing hands often (83.2%) was the most commonly reported preventive behavior [17]. As recommended by the World Health Organization, hand hygiene is one of the most effective ways to reduce the spread of pathogens and prevent infections [18]. Given the relatively low smartphone ownership (26.9%) among older adults, both online technology and real-life physical communication (e.g., phone calls, local newspapers, and radio) should focus on increasing public health outreach.

The present study has several strengths. First, it reached out to a unique population of the very old adults in the community using on-going longitudinal cohort resources. It might be difficult to enroll those elderly people into an online survey because of the relatively low use of smartphones and PCs in this age group. Second, a range of functional, mental, and behavioral factors was assessed before the COVID-19 outbreak using standardized and validated measures. There are important limitations to the present study. First, according to our study aims and eligibility criteria, we recruited community-dwelling, physically independent older adults for the KAWP. Thus, our respondents do not represent the general population with corresponding ages. Second, a selection bias might have occurred in the present survey conducted on 487 participants as a part of the KAWP study. In a questionnaire survey, it is important to achieve a high response rate within a pre-defined target population to ensure the validity of the results. Therefore, we made a substantial effort to achieve a high response rate of 89.2% in the present survey, and we believe this is a good indicator for valid results representing the study population. Moreover, characteristics of the present survey were similar to baseline characteristics of non-respondents of the KAWP cohort (Supplementary Table 1). Third, the present telephonic survey was conducted for a median of 2.2-years after the baseline examination; thus, the functional and psychosocial assessment may not reflect the respondents’ state at the time of the survey. Therefore, these results should be interpreted with caution. Forth, behavioral response to COVID-19 emergency may have been affected by the speed and scale of the spread of SARS-CoV-2 transmission. During the emergency period for the first wave, the maximum number of new COVID-19 patients per day was 18 (1.18 per 100,000 on April 11, 2020) [19]. When comparing our results to the findings of other studies, these limitations should also be taken into account.

## Conclusions

In conclusion, our data suggest that the very old adults in an urban community decreased their physical activities during the COVID-19 emergency, as a possible preventive behavior. Moreover, those having visual impairment were more likely to experience negative impacts of COVID-19 emergency in terms of physical activities. Additionally, in contrast to the high rate of face mask use, handwashing compliance was low, suggesting a need for public health awareness drives on the importance of handwashing via various platforms sources. The findings provide valuable data for designing preventive strategies to maintain the health and wellbeing of older adults who are at greater risk of both severe COVID-19 and the consequences of physical inactivity.

## Supplementary Information

**Additional file 1.** COVID-19-related Telephone Survey Sheet. Supplementary Table 1. Supplementary Table 2.

## Data Availability

The datasets analyzed in the current study will be available upon request with an appropriate research arrangement with approval of the Research Ethics Committee of Keio University School of Medicine for Clinical Research.

## Abbreviations

COVID-19: Coronavirus disease 2019
KAWP: The Kawasaki Aging and Wellbeing Project
OR: Odds ratio
CI: Confidence interval
SARS-CoV-2: Severe acute respiratory syndrome coronavirus 2
ADL: Activities of daily living
IADL: Instrumental activities of daily living
IQR: Interquartile range
MMSE: Mini-mental state examination
GDS-15: Geriatric depression scale-15
