# Supplementary material for: Behavioral changes and hygiene practices of older adults in Japan during the first wave of COVID-19 emergency
Source: BMC Geriatr. 2021 Feb 24;21:137. doi: 10.1186/s12877-021-02085-1 (PMC7903371; doi:10.1186/s12877-021-02085-1)
Supplement: Supplementary file 1 — Additional file 1. COVID-19-related Telephone Survey Sheet. Supplementary Table 1. Supplementary Table 2. [file 12877_2021_2085_MOESM1_ESM.docx]

Additional File 1

Behavioral changes and hygiene practices of older adults in Japan during the first wave of COVID-19 emergency

Yasumichi Arai^1†^, Yuko Oguma^2^, Yukiko Abe^1^, Midori Takayama^3^, Azusa Hara^4^, Hisashi Urushihara^4^, Toru Takebayashi^5^

^1^Centre for Supercentenarian Medical Research, Keio University School of Medicine, 35 Shinanomachi, Shinjuku-ku, Tokyo, 160-8582, Japan

^2^Sports Medicine Research Center, Keio University, 4-1-1 Hiyoshi, Kohoku-ku, Yokohama-shi,, Kanagawa, 223-8521, Japan

^3^Faculty of Science and Technology, Keio University, 4-1-1 Hiyoshi, Kohoku-ku, Yokohama-shi, Kanagawa, 223-8521, Japan

^4^Department of Drug Development and Regulatory Science, Faculty of Pharmacy, Keio University, 1-5-30 Shibakoen, Minato-ku, Tokyo, 105-8512, Japan

^5^Department of Preventive Medicine and Public Health, Keio University School of Medicine, 35 Shinanomachi, Shinjuku-ku, Tokyo, 160-85820, Japan

Contents:

1. COVID-19-related telephone survey sheet
2. Supplementary Table 1
3. Supplementary Table 2

**COVID-19-related Telephone Survey Sheet**

ID: Name:

Date of survey:

Respondent: Himself / Herself / Family (spouse/child)

**“I would like to ask you about the changes in the living conditions under the State of Emergency Declaration issued by the government on April 7 in response to the spread of the COVID-19 infection.”**

Q1. "Have you experienced any changes in your basic lifestyle (smoking, drinking, eating, sleeping)?"

No / Yes, changed

If Yes, “What was the biggest change (with regard to Q1)?”

(free response)

Q2. "Have you experienced any changes in the amount of time you spend exercising for your health, such as taking a walk or doing exercises?"

No / Decreased / Increased

If changed, “How much of your physical activity changed?”

(free response)

Q3. "Has your weight changed?"

No / Decreased / Increased / Unknown

If changed, “How much of your weight changed?”

(free response)

Q4. "Has the frequency of going out changed?

No / Decreased / Increased

(During the week under the state of Emergency declaration) “How often did you go out?” (free response)

Q5. "Do you take any precautions when you go out? (multiple choice)

No / Wear mask / Wash hands more / Avoid crowds / Monitor body temperature

Q6. "Has your mode of transportation when you go out changed?

No / Yes, changed

If changed, decrease / increase in private car / public transportation (buses and trains), taxies / bicycles

Q7. "Has the number of people you converse with changed, including face-to-face, telephone, and Internet conversations (SNS, chat)?

No / Decreased / Increased

If changed, decrease / increase in conversation with family / friends / neighbors / others (free response)

Q8. "Has your conversation time (with regard to Q7) changed?

No / Decreased / Increased

Q9. "Do you have a personal computer?"

No / Yes, my own / Yes, family own

Q10. "Do you have a smart phone?"

No / Yes, my own

Q11. "Has the frequency of visits to hospitals, clinics, and dentists you regularly see changed?

No / Decreased / Increased

If decreased, the reason is long-term prescription / phone or online consultations / others (free response)

Q12 (Open Question): “What was the most troubling or inconvenient factor for you under the State of Emergency Declaration due to the COVID-19 emergency”

(free response)

Optional: (If you don’t mind me asking) Has anyone close to you been infected with the new coronavirus, including you ?

No / Myself / Family (cohabitation) / Family (separation) / Friend / Others

(free response)

Thank you for your cooperation.

Supplementary Table 1. Baseline Characteristics between Participants and Non-participants to the telephonic COVID-19 telephonic Survey

| characteristics | Participants  (N=487) | Non-participants  (N=539) | P |
| --- | --- | --- | --- |
| Age at baseline | 87.0 (1.4) | 87.1 (1.4) | 0.608 |
| Sex (female), n % | 246 (50.5) | 267 (49.5) | 0.401 |
| Living alone | 130 (26.7) | 152 (29.2) | 0.210 |
| Widowed | 219 (45.2) | 236 (44.1) | 0.382 |
| MMSE | 26.1 (2.8) | 26.0 (2.9) | 0.668 |
| GDS | 3.2 (2.8) | 3.4 (2.7) | 0.244 |
| IADL | 4.9 (0.4) | 4.8 (0.5) | 0.302 |

Abbreviations: MMSE: Mini-Mental State Examination; GDS: Geriatric Depression Scale; IADL: Instrumental activities of daily living.

Supplementary Table 2. Factors associated with decreased frequency of going out, clinic/hospital/dentists visit, and conversation partners

|  | Decreased frequencies of going out | | | | |  | Decreased clinic/ hospital/ dentists visit | | | | |  | Decreased conversation partners | | | | |
| --- | --- | --- | --- | --- | --- | --- | --- | --- | --- | --- | --- | --- | --- | --- | --- | --- | --- |
|  | Crude | |  | Adjusted | |  | Crude | |  | Adjusted | |  | Crude | |  | Adjusted | |
| Characteristics | OR | 95%CI |  | OR | 95%CI |  | OR | 95%CI |  | OR | 95%CI |  | OR | 95%CI |  | OR | 95%CI |
| Age | 1.00 | (0.88-1.14) |  | 0.96 | (0.82-1.11) |  | 1.04 | (0.89-1.21) |  | 0.98 | (0.82-1.16) |  | 0.87 | (0.75-1.00) ^e^ |  | 0.85 | (0.73-1.003) |
| Sex (female) | 1.69 | (1.18-2.43) ^e^ |  | 1.54 | (0.95-2.51) |  | 1.84 | (1.20-2.83) ^e^ |  | 1.53 | (0.88-2.68) |  | 1.12 | (0.77-1.62) |  | 1.08 | (0.65-1.78) |
| high education | 0.80 | (0.56-1.15) |  | 0.78 | (0.52-1.19) |  | 0.88 | (0.58-1.35) |  | 0.90 | (0.56-1.46) |  | 0.95 | (0.65-1.38) |  | 0.92 | (0.60-1.42) |
| Living alone | 1.06 | (0.71-1.59) |  | 0.98 | (0.59-1.58) |  | 1.39 | (0.88-2.20) |  | 1.31 | (0.77-2.23) |  | 0.93 | (0.61-1.43) |  | 0.75 | (0.45-1.24) |
| Widowed | 1.24 | (0.86-1.77) |  | 1.05 | (0.65-1.69) |  | 1.50 | (0.98-2.29) |  | 1.04 | (0.61-1.78) |  | 1.26 | (0.87-1.83) |  | 1.42 | (0.87-2.31) |
| Current alcohol use | 0.84 | (0.58-1.21) |  | 1.01 | (0.67-1.54) |  | 0.74 | (0.48-1.15) |  | 0.89 | (0.55-1.45) |  | 1.07 | (0.73-1.56) |  | 1.16 | (0.75-1.78) |
| Current smoker | 0.39 | (0.15-0.98) ^e^ |  | 0.45 | (0.15-1.35) |  | 0.33 | (0.08-1.45) |  | 0.60 | (0.13-2.86) |  | 0.40 | (0.13-1.22) |  | 0.46 | (0.12-1.72) |
| Overweight | 1.03 | (0.68-1.55) |  | 0.96 | (0.61-1.50) |  | 1.14 | (0.71-1.83) |  | 1.13 | (0.68-1.88) |  | 1.27 | (0.84-1.93) |  | 1.44 | (0.91-2.28) |
| High self-rated health^a^ | 0.96 | (0.67-1.38) |  | 0.81 | (0.54-1.21) |  | 0.91 | (0.59-1.40) |  | 0.80 | (0.50-1.28) |  | 0.89 | (0.61-1.30) |  | 0.87 | (0.57-1.33) |
| MMSE (≤23) | 0.51 | (0.31-0.84) ^e^ |  | 0.58 | (0.33-0.99)^e^ |  | 1.09 | (0.62-1.92) |  | 1.15 | (0.62-2.12) |  | 0.63 | (0.37-1.09) |  | 0.60 | (0.32-1.10) |
| GDS (≥5) | 0.93 | (0.61-1.41) |  | 0.92 | (0.58-1.47) |  | 0.89 | (0.54-1.46) |  | 0.78 | (0.44-1.36) |  | 1.01 | (0.65-1.55) |  | 1.06 | (0.65-1.72) |
| IADL (≤4) | 1.11 | (0.65-1.92) |  | 1.14 | (0.61-2.14) |  | 1.42 | (0.78-2.57) |  | 1.50 | (0.68-3.15) |  | 1.27 | (0.74-2.20) |  | 1.17 | (0.62-2.22) |
| Chronic conditions ≥5 | 1.28 | (0.89-1.85) |  | 1.28 | (0.86-1.91) |  | 1.05 | (0.69-1.61) |  | 1.12 | (0.71-1.78) |  | 0.88 | (0.61-1.29) |  | 0.83 | (0.55-1.25) |
| Hearing impairment | 0.79 | (0.40-1.56) |  | 0.66 | (0.31-1.44) |  | 0.28 | (0.08-0.92) ^e^ |  | 0.31 | (0.09-1.06) |  | 0.77 | (0.37-1.60) |  | 0.72 | (0.32-1.66) |
| Visual impairment | 0.95 | (0.63-1.45) |  | 1.02 | (0.64-1.61) |  | 1.03 | (0.63-1.68) |  | 1.09 | (0.64-1.86) |  | 1.28 | (0.84-1.97) |  | 1.21 | (0.75-1.94) |
| Engagement in physical activity^b^ | 1.12 | (0.77-1.64) |  | 1.20 | (0.79-1.84) |  | 1.14 | (0.73-1.77) |  | 1.26 | (0.77-2.04) |  | 1.67 | (1.13-2.47) ^e^ |  | 1.90 | (1.23-2.93) ^e^ |
| Frequent community interaction^c^ | 0.94 | (0.55-1.59) |  | 0.99 | (0.55-1.82) |  | 0.83 | (0.43-1.59) |  | 0.87 | (0.44-1.75) |  | 0.48 | (0.26-0.90) ^e^ |  | 0.43 | (0.21-0.88) ^e^ |
| Smartphone ownership | 1.73 | (1.14-2.63) ^e^ |  | 1.82 | (1.15-2.88) ^e^ |  | 0.93 | (0.58-1.51) |  | 0.98 | (0.58-1.65) |  | 0.93 | (0.61-1.42) |  | 0.91 | (0.57-1.45) |

Abbreviations: OR: Odds ratio; CI: Confidence interval; MMSE: Mini-Mental State Examination; GDS: Geriatric Depression Scale; IADL: Instrumental activities of daily living.

^a^ Those who reported very good/good versus others (reference).

^b^ Those who reported upper tertile of physical activity versus others (reference).

^c^ Those who reported every day versus others (reference).

^d^ Those who reported everyday versus others (reference).

^e^ P<0.05.
